# Hydromorphone-Induced Tactile Hallucinations: Rare Opioid Side Effect

**DOI:** 10.7759/cureus.13622

**Published:** 2021-02-28

**Authors:** Miki Kiyokawa, William F Haning

**Affiliations:** 1 Psychiatry, University of Hawaii John A. Burns School of Medicine, Honolulu, USA; 2 Internal Medicine, University of Hawaii John A. Burns School of Medicine, Honolulu, USA

**Keywords:** hydromorphone, opioid side effects, tactile hallucination, formication

## Abstract

Opioids are strong analgesics widely employed to treat various types of pain. In 2018, an estimated 168 million opioid prescriptions were dispensed in the United States. Opioids carry a number of side effects and up to 80% of patients treated with opioids experience a minimum of one adverse event. Although uncommon, hallucinosis is an effect experienced with opioids, which may be under-reported and attributed to underlying psychiatric disease rather than to the side effects of the opioid itself. Most of the opioid-induced hallucinoses reported are auditory and visual, and rarely tactile. Although opioid medication prescribing is decreasing in the United States, considering the continued opioid epidemic and deaths related to overdose, it is important for physicians to be aware of this potential adverse effect of opioids in isolation. We present a case of oral hydromorphone causing visual and tactile hallucinations. Discontinuing hydromorphone led to immediate cessation of the patient’s psychotic signs and symptoms. To our knowledge, this is the first description of the use of hydromorphone resulting in tactile hallucinations.

## Introduction

In 2018, approximately 168 million opioid prescriptions were dispensed in the United States (US). Although the overall national opioid prescription rate has declined since 2012, the US continues to exceed all other nations in daily doses of prescription opioids per million inhabitants [1,2].

Up to 80% of patients treated with opioids experience a minimum of one adverse event [3]. Opioid-induced hallucinations are an uncommon but significant adverse effect, which may be attributed to underlying psychiatric disease and consequently be under-reported, rather than recognized as side effects of the opioid itself. In some cases, patients feel uncomfortable speaking-up about experiences for fear of being considered psychologically unstable [4,5]. There are citations reporting opioid hallucinations, mostly auditory and visual, but rarely of the tactile nature that our patient experienced [4]. As far as we are aware, there is no report of this phenomenon in association with hydromorphone. We report a case of a young woman who experienced multiple visual hallucinations and an episode of tactile hallucination manifested shortly after receiving oral hydromorphone. Both visual and tactile hallucinations ceased following the discontinuation of the hydromorphone.

## Case presentation

Initial hospitalization

A young intravenous heroin user in her 30s with a history of seizure disorder (on levetiracetam and lamotrigine), depression, and anxiety was seen in the emergency department (ED) for lethargy and fever. The physical exam was notable for left hemiparesis and several injection marks on the arms and feet. The patient’s urine drug screen was positive for opiates. Computerized tomography and magnetic resonance imaging of the head showed multiple lesions suggesting embolic infarcts. A subsequent echocardiogram showed a 10-mm variegated density suspicious for vegetation.

The patient eventually underwent valve replacement surgery and oral hydromorphone 2mg every four hours as needed for pain was started, of which she required no more than four doses per day. The patient was receiving daily non-pharmacological management for pain and anxiety from the hospital psychiatric team and she enjoyed good family support, with someone always present at the bedside. Hydromorphone was discontinued by the 10th postoperative day and was replaced with daily buprenorphine/naloxone sublingual (2mg/0.5mg) to treat her opioid use disorder. The patient was eventually discharged to a physical rehabilitation hospital for her left hemiparesis, with her outpatient antiseizure medications, aspirin, and buprenorphine/naloxone.

Second hospitalization

Shortly thereafter, the patient was readmitted due to pericardial effusion. After a surgical procedure, oral hydromorphone 2mg every four hours as needed for pain was initiated. She required no more than four doses a day. All outpatient medications were continued except for aspirin and buprenorphine/naloxone. As in the first hospitalization, the inpatient psychiatry team was involved with the non-pharmacological treatment of pain and anxiety.

On postoperative day nine, hydromorphone was discontinued, and buprenorphine/naloxone (2mg/0.5mg) was restarted. The patient was discharged to the rehabilitation hospital with oral colchicine and ibuprofen for the pericardial effusion, outpatient antiseizure medications, and buprenorphine/naloxone (2mg/0.5mg).

A week following discharge, the patient’s buprenorphine/naloxone dose was increased to 4mg/1mg per day.

Third hospitalization

A month later, the patient was again hospitalized with pericardial effusion. The patient received fentanyl and midazolam during the surgical procedure. The inpatient psychiatry team followed the patient for non-pharmacological management for anxiety and pain.

On the second hospital day, oral hydromorphone 2mg every four hours as needed for pain from the surgical procedure was started and the patient received a total of two doses that day. All outpatient medications, including buprenorphine/naloxone 4mg/1mg for opioid use disorder, were continued. In the evening, prednisone 20mg daily was started for postpericardiotomy syndrome.

On the early morning of the third hospital day, the patient received a dose of hydromorphone. In the late morning, the patient received the second dose of hydromorphone for the day and shortly after, the patient reported experiencing bugs crawling on her forehead and in her hair, which resolved after an interval of 30 to 60 minutes. Responding to the possibility of tactile hallucinations, the team followed careful scrutiny of her bedclothes and room, a complete physical examination, and selected laboratory studies including complete blood count with differentials, glucose, renal profile (includes electrolytes), hepatic function profile, all of which proved unremarkable. Except for the perceptual disturbance, a mental status examination disclosed no abnormality and the patient remained alert and oriented to person, place, time and situation before and after the incident. Family members who were at her bedside daily expressed no concern about the patient’s mentation other than the formication. 

Upon further interview, the patient reported seeing two to three black dime-sized insect shapes that she described as a “beetle” and a “fly” at the corner of her visual field since the first hospitalization. These creatures appeared to crawl along the bed rails and at times to fly. The patient’s relative who was at the bedside daily reported their arguing about the phenomena; the patient thought that the hospital was infested with bugs, leading to arguments between them surrounding reality. The relative realized that during the second hospitalization, the patient routinely complained about the bugs shortly after taking each dose of oral hydromorphone that the complaints would resolve in a matter of hours. No change in the patient’s mental status or behavior was noted by the relative and she was noted to talk, act, and respond at her baseline except for complaining of the bugs. The relative immediately addressed the concerns to one of the non-physician health care professionals; however, none of the physicians was informed. The tactile sensation of bugs crawling was not experienced until the third morning of the third hospitalization. The patient reports no history of hallucinations prior to this series of hospitalizations.

Hydromorphone was discontinued and oral oxycodone 10mg every four hours as needed for pain was initiated. She continued to receive buprenorphine for opioid use disorder, prednisone for her postpericardiotomy syndrome, and midazolam and fentanyl for subsequent surgical procedures. The patient never saw bugs or experienced formication after the hydromorphone was discontinued. The patient was discharged with the same regimen as in the second hospitalization along with prednisone 20mg daily. Buprenorphine/naloxone 4mg/1mg sublingual had been maintained throughout the hospitalization and was continued as an outpatient. The patient was followed up as an outpatient at one week and one month following discharge without recurrence of hallucinations.

## Discussion

We report the case of a young woman who experienced multiple visual hallucinations and an episode of tactile hallucination related to oral hydromorphone administration. Hallucinations can occur due to multiple factors, especially in an inpatient setting. Potential contributing factors for our patient include delirium, underlying psychiatric disorder, and medications.

Hallucinations associated with delirium are usually seen in those who are critically ill and in patients who are in acute substance intoxication or withdrawal [6]. With our patient, findings consistent with delirium, intoxication, or withdrawal did not emerge during the three hospitalizations. Her last alcohol intake or substance use was almost three months prior to the episode of formication. On the day the patient experienced formication, a thorough evaluation did not indicate an origin for the sensations, and her mental status exam was otherwise normal where she remained alert and oriented to person, place, time, and situation before and after the incident.

The patient’s reported anxiety and depression were also considered possible contributors to the hallucinosis; however, the patient denied experiencing hallucinations previously. The patient’s anxiety and depression were treated non-pharmacologically by the inpatient psychiatry team, with improvement during the hospitalization, hence, are less likely to be the culprit of hallucination.

Some of the medications administered to our patient have been associated with hallucinations. Potentially causative medications include levetiracetam [7,8] and lamotrigine [9], the patient’s long-term outpatient medications for her seizure disorder; midazolam and fentanyl [10], given during the operative procedures; glucocorticoids [11]; nonsteroidal anti-inflammatory drugs (NSAIDs) [12]; and buprenorphine [13]. Administration of the above medications however did not correlate with symptom development or cessation in this patient. Both onset and resolution of the psychotic events were clearly correlated with the interval of hydromorphone use.

The literature contains reports of opioid-related hallucinations, mostly auditory and visual but rarely tactile. The majority of the hallucinations reported is from morphine and occurred on high-dose opioid regimens [4]. In the study of Bruera, four cases of visual hallucinations were reported associated with high doses of hydromorphone in patients who had advanced cancer and without pre-existing cognitive impairment. The hallucinations resolved after changing to a different opioid and with concomitant administration of haloperidol [14]. In our patient, cessation of hydromorphone led to resolution of the hallucinations, requiring no antipsychotic medications.

Our patient received solely hydromorphone at the time she began to experience visual hallucinations (during the first and second hospitalizations). Her maximum dose consumption was 32 morphine milligram equivalents (MME) per day. During the third hospitalization when she experienced tactile hallucinations, she was receiving maintenance buprenorphine/naloxone 4mg/1mg (120 MME) and hydromorphone (16 MME the day before and 16 MME the day of formication), a significantly higher MME than during the first and second hospitalization. Perhaps hallucinations induced by hydromorphone, alone or in combination with buprenorphine, may be dose related and may have added to the complexity of the hallucinatory experience [4,13]. We believe it worth noting that when the patient was receiving oxycodone and buprenorphine in the absence of hydromorphone, there was no recurrence of hallucinations. The patient remains hallucination-free as an outpatient while receiving buprenorphine/naloxone (4mg/1mg).

## Conclusions

Opioids are widely used in the US. There are numerous side effects including hallucinations, and the complexity of the hallucination may be related to the dose. Unfortunately, opioid-induced hallucinations may be under-reported or attributed to underlying psychiatric disease rather than to the opioid itself. Hallucinations from opioids are commonly visual or auditory, and tactile hallucinations are very rare. To the best of our knowledge, there have been no reports of tactile hallucinations associated with hydromorphone. With the continuing practice of opioid prescription, it is important for physicians to recognize this potential adverse effect of hydromorphone and possibly of related opioids.
